# Transcriptomics reveal the involvement of reactive oxygen species production and sequestration during stigma development and pollination in *Fraxinus mandshurica*

**DOI:** 10.48130/forres-0024-0011

**Published:** 2024-04-23

**Authors:** Shuqi Wang, Shun Yang, Bello Hassan Jakada, Hongtao Qin, Yaguang Zhan, Xingguo Lan

**Affiliations:** Key Laboratory of Saline-Alkali Vegetation Ecology Restoration, Ministry of Education, College of Life Sciences, Northeast Forestry University, Harbin 150040, China

**Keywords:** *Fraxinus mandshurica*, Stigma development, Pollination, Pollen germination, Transcriptome, Reactive oxygen species

## Abstract

Stigma development and successful pollination are essential for the continuous existence of flowering plants. However, the specific mechanisms regulating these important processes are not well understood. In this study, we investigated the development of the stigma in *Fraxinus mandshurica*, dividing it into three stages: S1, S2, and S3. Transcriptome data were used to analyze the gene expression patterns across these developmental stages, and 6,402 genes were observed to exhibit variable expression levels. Our analysis revealed a significant enrichment of pathways related to reactive oxygen species (ROS) and flavonoids, as indicated by the Kyoto Encyclopedia of Genes and Genomes enrichment analysis of the differentially expressed genes. Further examination by cluster analysis and quantitative polymerase chain reaction revealed that 58 genes were associated with ROS synthesis and seven genes were linked to flavonoid synthesis during the S2 and S3 stages. ROS accumulated during stigma development, which decreased rapidly upon pollen germination and pollen tube elongation, as confirmed by H_2_DCFDA staining. Moreover, ROS levels in mature stigmas were reduced by treatment with ROS scavengers, such as copper (II) chloride, sodium salicylate, and diphenyleneiodonium, an inhibitor of NADPH oxidases, which enhanced pollen adhesion and germination. These findings suggest that the balance between ROS production and sequestration plays a critical role in regulating stigma development and pollen germination in *Fraxinus mandshurica*.

## Introduction

Gametophyte development and pollination are critical in the life cycle of flowering plants, as well as fruit production^[1,2]^. Many genes, proteins, and pathways regulate pollination in plants. However, in certain plant species, these genes, proteins, and pathways undergo distinct changes in both the stigma and pollen, establishing a barrier to reproduction^[3]^. The stigma receives pollen in the process of pollination, and based on the secretory substances on the stigma, the stigma can be divided into wet and dry. Dry stigmas, found in Brassicaceae and Poaceae families, are characterized by a waxy proteinaceous cuticle covering their surface^[4]^. Conversely, wet stigmas, found in Liliaceae, Solanaceae, and Rosaceae families, are saturated with water droplets and lipids, which facilitate pollen adhesion and hydration^[5−7]^. Lipids are also important for pistil fertility, with cis-unsaturated triacylglycerides playing a critical role^[8]^.

While the adhesion and hydration of pollen is less regulated in wet stigmas, dry stigmas show selective pollen acceptance due to the existence of surface barriers that promote pollen tube penetration^[9]^. During pollination, pollen comes in contact with the mature stigma, triggering changes in signaling molecules and critical metabolites^[10]^. In dry stigma, pollen capture mainly relies on the physical and chemical properties of the pollen surface, and upon the capture of compatible pollen, POLLEN COAT PROTEIN B-CLASS PEPTIDE (PCP-bs) binds to FERONIA (FER) and ANJEA (ANJ), leading to a reduction in ROS levels^[11]^. Subsequently, an increase in the second messenger Ca^2+^ promotes pollen germination^[12−14]^. In flowering plants, reactive oxygen species (ROS) play an important role in the development of male and female gametophytes and the interaction between pollen and stigma^[15]^.

After the stigma recognizes the pollen, the downstream signal in the stigma responds quickly. The downstream protein of the S-LOCUS RECEPTOR KINASE (SRK), ARM-REPEAT CONTAINING 1 (ARC1), is not phosphorylated, allowing EXOCYST70A1 (EXO70A1), GLYOXALASE1 (GLO1), and PHOSPHOLIPASE D1 (PLD1) to function normally^[16]^. EXO70A1 promotes vesicle fusion and secretion, which are crucial for pollen hydration^[17]^, while the lyase GLO1 regulates methylglyoxal levels in the cytoplasm, thereby reducing its content due to glucose metabolism during pollination^[18]^. PLD1 produces phosphatidic acid, promoting vesicle fusion in-stigma mastoid cells and facilitating exocytosis for pollen germination^[16]^.

In recent years, the rapid development of omics technologies, such as RNA sequencing and proteomics, has enabled researchers to explore the mechanisms underlying gametophyte development and pollination. An analysis of stigmatic exudates from *Lilium longiflorum* and *Olea europaea* identified 51 and 57 proteins, respectively, the majority of which were newly discovered, and many of which were implicated in regulating pollen growth^[19]^. In another study, mass spectroscopy coupled with 3D gel-based techniques identified more than 2,100 triticale stigma proteins involved in stigma development, pollen–stigma interactions, and environmental stress response^[20]^. Integration of PacBio SMRT-seq and Illumina RNA-seq technologies in ornamental kale resulted in the construction of a transcriptome database covering the various stages of stigma development and leading to the discovery of novel genes, transcripts, non-redundant transcripts, alternatively spliced variants, complete open reading frame sequences, simple sequence repeats, and long non-coding RNAs, all of which are important for the biology of the stigma^[21]^. Thus, the development of omics technologies can provide new ideas in *Fraxinus mandshurica* research.

*Fraxinus mandshurica* (*F. mandshurica*) is a large deciduous tree belonging to the Oleaceae family, valued for its monetary worth and medical properties^[22]^. Propagated by seed, this plant has a low seed-setting rate due to various physiological factors that influence gametophyte development, pollination, and fertilization^[23]^. Understanding how *F. mandshurica* regulates and optimizes gene expression during stigma development could be instrumental in preserving its reproductive capability to ensure successful cross-pollination and seed formation for its conservation. Despite its importance, there is a lack of transcriptional profiling studies exploring stigma development in *F. mandshurica*. To address this gap, we use RNA-sequencing (RNA-seq) to compare the transcriptomic profiles of *F. mandshurica* stigma at various stages of development, and this study serves as an avenue for improvement of *F. mandshurica* cultivation and conservation.

## Materials and methods

### Plant material and morphological analysis

*F. mandshurica* from the Northeast Forestry University (Harbin, China) experimental forest was used as the experimental material. The stigmas of *F. mandshurica* were collected at different stages of development. In the initial stage (S1), approximately 3,000 stigmas were collected per sample. These stigmas were characterized as small and ellipsoidal, without bifurcation, and measuring less than or equal to 1 mm in length. In the subsequent stage (S2), approximately 2,000 stigmas were collected as they reached a size of 1.5–2.0 mm and started to show signs of bifurcation. In the final stage (S3), approximately 1,500 stigmas were collected per sample. At this stage, the stigmas had become bifid, with branches exceeding 2.5 mm in length, resembling sheep horns in shape, and displaying white coloration along the edges. Three biological replicates were collected for each stage and labeled as S1, S2, and S3. The samples were flash-frozen using liquid nitrogen and stored at –80 °C for RNA extraction.

### RNA isolation and RNA sequencing

The stigmas of *F. mandshurica* were processed for RNA isolation using TRNzol Universal Reagent (Tiangen, Beijing, China), according to the manufacturer's protocol. The quantity and quality of the RNA were assessed using spectroscopy (NanoDrop2000, Thermo Fisher Scientific, Waltham, MA, USA) and 1.5% agarose gel electrophoresis, respectively. The Illumina HiSeq 2000 platform (San Diego, CA, USA) was used to sequence the 9 RNA samples (three replicates for each stage). RNA-seq by expectation-maximization and fragment per kb of transcript per million fragments mapped were used to calculate the gene transcript levels in different stages of stigma development^[24]^. DESeq2 (https://rdrr.io/bioc/DESeq2/src/R/core.R) was employed to extract differentially expressed genes (DEGs) with criteria set to |log2Fold Change| ≥1 and false discovery rate <0.05^[25]^.

### RT-qPCR analysis

Primer-Blast (www.ncbi.nlm.nih.gov/tools/primer-blast/index.cgi) was used to design primers for quantitative polymerase chain reaction (qPCR). cDNA synthesis was performed using TransScript One-Step cDNA Synthesis and gDNA Removal SuperMix (TransGen, Beijing, China), while the LightCycler480 system (Roche, Basel, Switzerland) was employed for qPCR using SYBR qPCR Mix (TransGen, Beijing, China). Relative gene expression levels were calculated using the 2^−ΔΔCᴛ^ method^[21]^, with *FmTUB* serving as the housekeeping gene for normalization. The experiment was carried out with three biological and three technical replicates.

### ROS detection in stigmas

The ROS levels in the stigmas of *F. mandshurica* were measured using 2,7-dichlorodihydrofluorescein diacetate (H_2_DCFDA). The stigmas were immersed in 10 mM MES, 5 mM KCl, 50 mM CaCl_2_, pH 6.15, containing 50 μM H_2_DCFDA, and then rinsed at least three times before observation, each group includes 13 stigmas. The average signal intensity in regions of interest was quantified using ImageJ software (National Institutes of Health, Bethesda, MD, USA). For comparison, the S1 group is defined as the control group, ROS levels in control stigmas were set to 1.

### Excised stigma-feeding assays and pollination visualization

The excised stigmas were immersed into 5 mM CaCl_2_, 5 mM KCl, 0.01% H_3_BO_3_, 1 mM MgSO_4_·7H_2_O, 10% sucrose, 0.8% agarose, pH 7.5 for 1 h. Initially, mature stigmas were treated with copper (II) chloride (CuCl_2_), sodium salicylate (Na-SA), or an inhibitor of NADPH oxidases, diphenyleneiodonium chloride (DPI). The mock-treated group of CuCl_2 _and Na-SA was treated with ddH_2_O. The mock-treated group of DPI was treated with 0.1% DMSO. The stigmas were placed in a chamber at a temperature of 22.5 °C and a humidity of 45% for 60 min. Subsequently, the stigmas were manually pollinated once and then placed in a chamber under identical temperature and humidity for 30, 60, and 120 min. Following this, an aniline blue stain was applied, and pollen adhesion and germination were observed using a Leica DM4 B microscope (Wetzlar, Germany).

### Statistical analysis

Data on ROS, pollen number, and pollen tube number are presented as bar graphs generated in GraphPad Prismv 8.0.1. The dots in bar graphs denote individual data points ± SEM for all stigmatic ROS data and all the pollen tube growth data. Student's t-test was used to determine the statistical significance, asterisks or ns directly above the data bars indicate a significant difference (*p < 0.05, **p < 0.01, ***p< 0.001).

## Results

### Distinct developmental stages are observed in the *F. mandshurica* stigma

**Figure 1:**
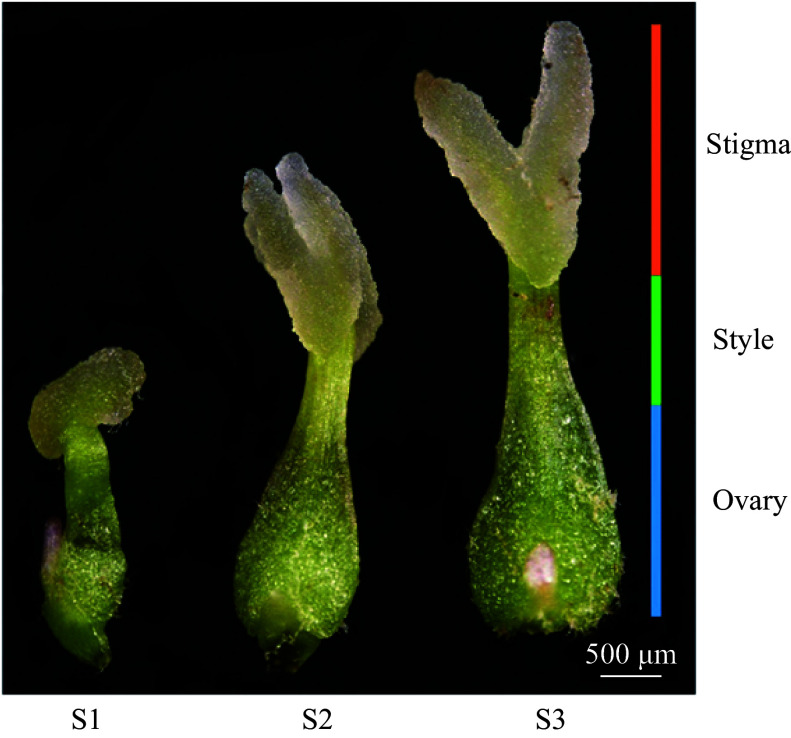
Morphological stages of stigma development in *F. mandshurica*. From left to right: S1, S2, and S3 stages, as observed by stereomicroscopy. The orange bar corresponds to the stigma, the green bar to the style, and the blue bar to the ovary. Scale bar = 500 µm.

Three distinct developmental stages were observed in *F. mandshurica* stigmas by stereomicroscopy. During the S1 stage, the stigma was characterized as small and ellipsoidal, with no observable bifid structure. The stigma appeared light yellow and measured 1 mm or less in length. In the S2 stage, the stigma elongated and began to show signs of bifurcation. Its length increased to 1.5–2.0 mm, and the yellow color along the edges of the stigma began to fade to white. By the S3 stage, the stigma displayed a bifid structure, with branches exceeding 2.5 mm in length, resembling sheep horns in shape, and displaying white coloration along the edges (Fig. 1).

### Differential expression analysis identified enriched pathways during stigma development in *Fraxinus mandshurica*

Raw reads of 74.23–90.44 million basepairs were generated from nine RNA samples using the Illumina HiSeq platform (Supplemental Table S1), resulting in 70.94–86.85 million clean reads per library, accounting for 95.72% of total reads (Supplemental Fig. S1a). In total, 41,286 unigenes were successfully annotated in at least one database. The quantity and quality of the sequencing data satisfied the requirements for further downstream analysis.

**Figure 2:**
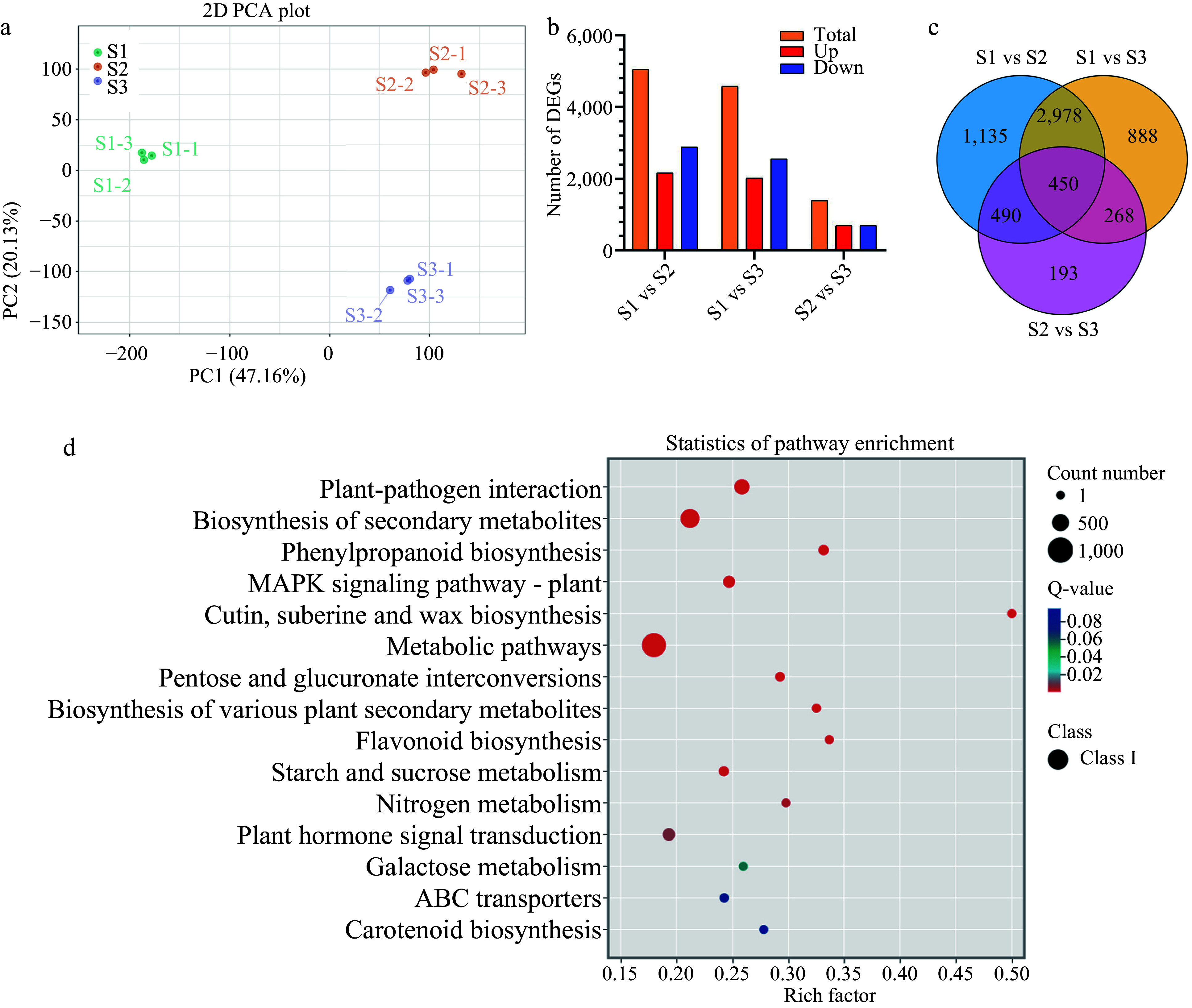
Statistical analysis of differentially expressed genes (DEGs) related to stigma development in *F. mandshurica*. (a) Principal component analysis (PCA) of transcriptome data during stigma development. (b) Summary of DEG results. Red and blue indicate upregulated and downregulated genes, respectively. In each comparison, orange indicates the total. (c) Venn diagram of DEGs in three comparison groups. (d) KEGG enrichment analysis of all DEGs.

Subsequent analysis was conducted based on |log2Fold Change| ≥1 and FDR <0.05 as significance cut-offs. The principal component analysis (PCA) of gene expression levels revealed minimal differences among the three biological replicates compared to between the samples (Fig. 2a). Differential expression analysis during stigma development identified 5,053 (2,171 upregulated and 2,882 downregulated), 4,584 (2,023 upregulated and 2,561 downregulated), and 1,401 (702 upregulated and 699 downregulated) DEGs between S1 vs S2, S1 vs S3, and S2 vs S3 libraries, respectively (Fig. 2b). Furthermore, 450 DEGs were common to all libraries (Fig. 2c), potentially functioning in all stages of stigma development. Similarly, 3,460 transcription factors (TFs) were identified and categorized into 94 TF families, with AP2/ERF-ERF (228 genes), bHLH (209 genes), MYB (200 genes), and C2H2 (175 genes) families being the largest (Supplemental Fig. S1b).

Gene Ontology enrichment analysis revealed that DEGs were predominantly associated with the biological process category (cellular and metabolic processe), cellular component category (cellular and anatomical entity), and molecular function category (binding and catalytic activity) (Supplemental Fig. S1c–e).

In each comparison group (S1 vs S2, S2 vs S3, S1 vs S3), the top 15 pathways with the smallest q-values in the KEGG analysis were considered significant (Supplemental Fig. S1f–h). To investigate the biological and metabolic processes involved in stigma development, we used the KEGG database to annotate all DEGs. Among the 15 pathways showing enrichment, a strong presence of plant–fungal interactions were observed, followed by secondary metabolite biosynthesis, carotenoid biosynthesis, flavonoid biosynthesis, metabolic processes, plant–hormone signaling, and MAPK signaling (Fig. 2d).

### Expression of ROS-related genes is significantly increased at S2 and S3 stages in the *F. mandshurica* stigma

In plants, ROS are mainly produced within subcellular compartments, thus requiring efficient mechanisms to regulate their levels. Our analysis of RNA-seq data focused on genes associated with ROS production and sequestration. A total of 58 genes were differentially expressed, including six enzymes related to ROS production and seven enzymes related to ROS elimination.

**Figure 3:**
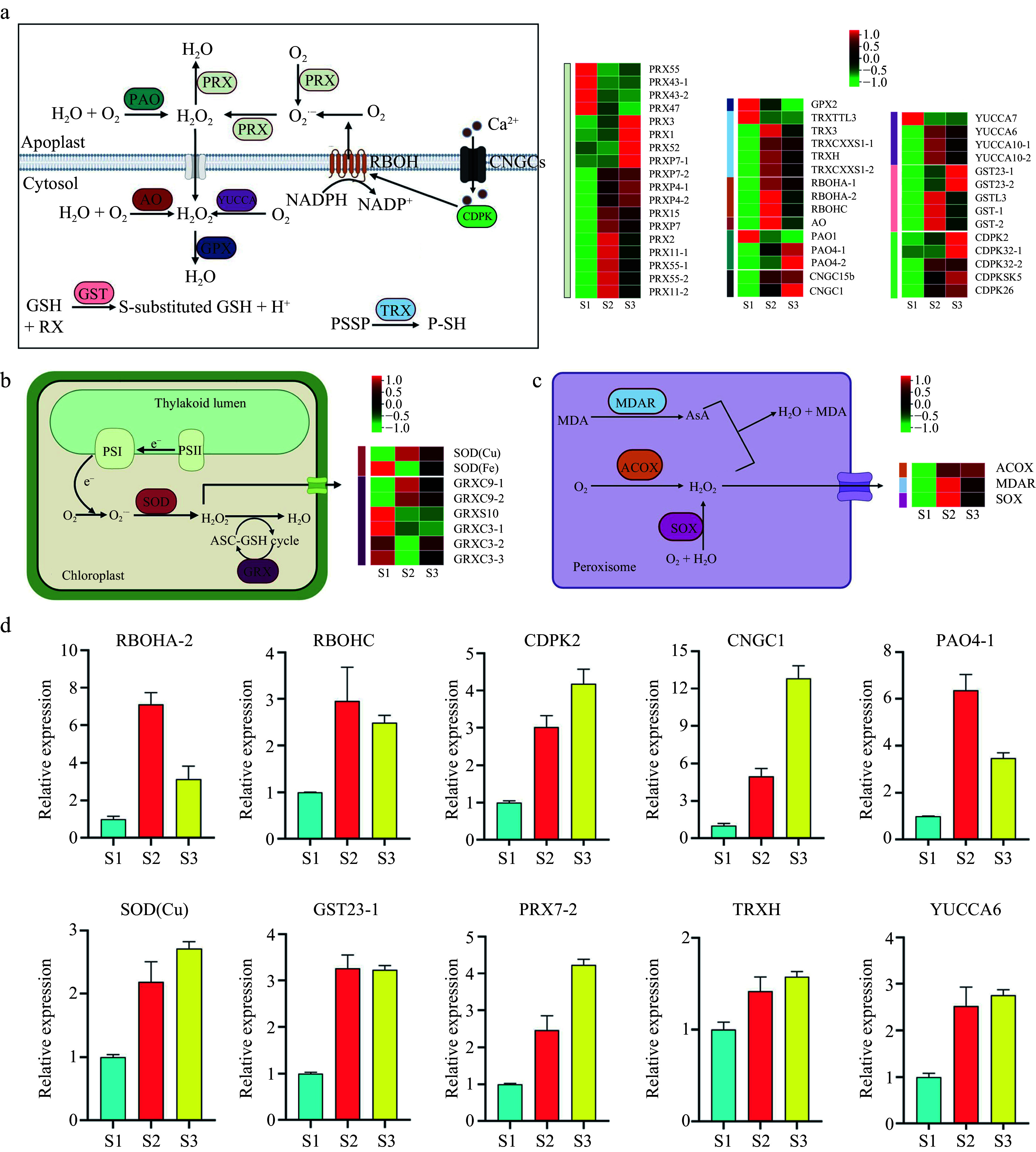
Pathway cluster analysis and quantitative polymerase chain reaction (qPCR) validation of selected genes related to ROS production and sequestration. (a) ROS production and sequestration in the apoplast and cytosol. (b) ROS production and sequestration in the chloroplast. (c) ROS production and sequestration in the peroxisome. (d) qRCR analysis of selected genes. ACOX, Acyl-CoA oxidase; AO, Aldehyde oxidase; AsA, Ascorbic acid; CDPK, Calcium-dependent protein kinase; CNGC, Cyclic nucleotide-gated channel; GPX, Glutathione peroxidase; GRX, Glutaredoxin; GST, Glutathione S-transferase; MDAR, Monodehydroascorbate reductase; MDA, Malondialdehyde; PAO, Polyamine oxidase; PRX, Peroxidase; RBOH, Respiratory burst oxidase homolog; SOD, Superoxide dismutase; SOX, Sarcosine oxidase; TRX, Thioredoxin.

Between the apoplast and the cytosol, two *RESPIRATORY BURST OXIDASE HOMOLOGS* (RBOH: NADPH oxidase) were highly expressed in the S2 stage, with one additionally showing increased expression in the S2 and S3 stages. Three *POLYAMINE OXIDASES* (PAO) were also differentially expressed, with two showing high expression in the S3 stage and one in the S1 stage. *ALDEHYDE OXIDASE* (AO) demonstrated elevated expression in the S2 stage, while *YUCCA* exhibited increased expression in S2 and S3 stages. Notably, most genes encoding ROS scavenging enzymes, such as *GLUTATHIONE PEROXIDASE* (GPX), *GLUTATHIONE S-TRANSFERASE* (GST), *THIOREDOXIN* (TRX), and *PEROXIDASE* (PRX), were highly expressed in the S2 and S3 stages (Fig. 3a).

In the chloroplast, one *SUPEROXIDE DISMUTASE* (SOD) gene was highly expressed in S2 and S3 stages, while two *GLUTAREDOXINS* (GRX) were highly expressed in S2 and S3 stages (Fig. 3b), all of which are known to scavenge ROS. Similarly, in the peroxisome, the expression of *ACYL-COA OXIDASE* (ACOX) and *SARCOSINE OXIDASE* (SOX) increased across the three developmental stages, and *MONODEHYDROASCORBATE REDUCTASE* (MDAR) was highly expressed in S2 and S3 stages (Fig. 3c). Ten genes were selected for qPCR, and the findings aligned with those of RNA-seq (Fig. 3d).

### Genes associated with flavonoid synthesis may be specific to S2 and S3 stages in the *F. mandshurica* stigma

**Figure 4:**
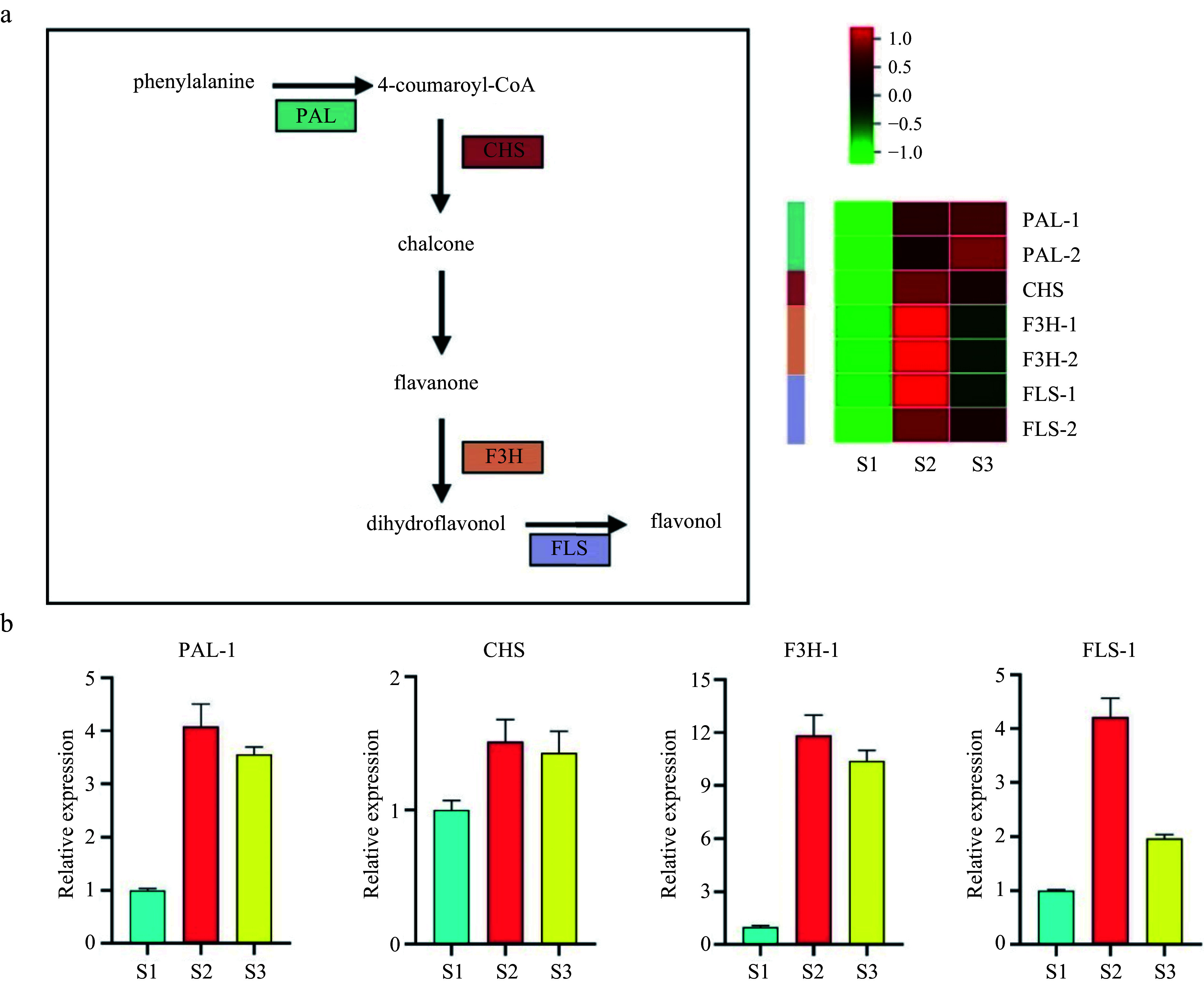
Validation of potential genes associated with flavonoid synthesis by cluster analysis and quantitative polymerase chain reaction (qPCR). (a) Pathway and heatmap of candidate genes showing fold changes. (b) qPCR validation of flavonoid synthesis-related genes. PAL, Phenylalanine ammonia-lyase; CHS, Chalcone synthase; F3H, Flavanone 3P-hydroxylase; FLS, Flavonol synthase.

The KEGG analysis revealed a significant enrichment of flavonoid synthesis pathways across the three developmental stages. Our analysis of the RNA-seq data identified seven genes involved in flavonoid synthesis, and the levels of these genes were increased in S2 and S3 stages. To confirm the reliability of our transcriptome data, we conducted qPCR on four flavonoid synthesis genes. The results revealed high expression levels of *PHENYLALANINE AMMONIA-LYASE* (PLA), *FLAVANONE 3P-HYDROXYLASE* (F3H), *CHALCONE SYNTHASE* (CHS), and *FLAVONOL SYNTHASE* (FLS) in S2 and S3 stages (Fig. 4a, b). Taken together, these findings indicate that genes involved in flavonoid synthesis are specific to the S2 and S3 stages of stigma development and may play a role in pollen–stigma interactions.

### ROS accumulation decreases after pollination

**Figure 5:**
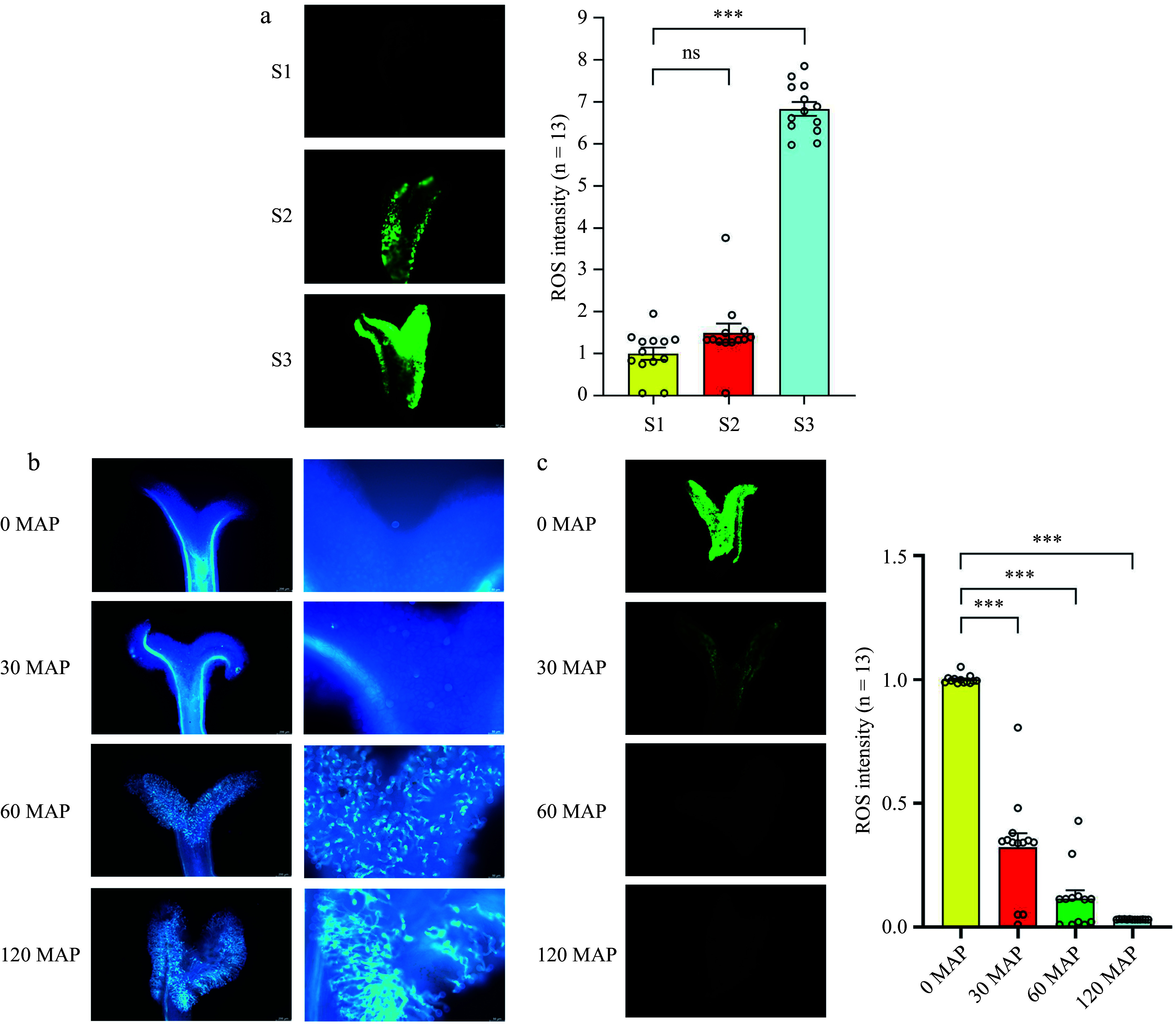
Accumulation of ROS in the stigma, followed by a reduction in ROS levels after pollination. (a) Detection of ROS during stigma development. (b) Observation of pollen grains under blue light after staining with aniline blue. From left to right: pollen grain number and pollen tube germination. (c) H_2_DCFDA staining of ROS levels at different time points after pollination. ROS signals were measured using ImageJ (the average signal in S1 stigmas is set at 1 for comparison). Data are expressed as ± SEM. Each data point is indicated by a dot, and (n) denotes stigma number. Asterisks or not significant (ns) above the error bars indicate significance level compared with the data bar on the left (Student's t-test; ***p < 0.001).

To evaluate the ROS levels across the three developmental stages, we stained the stigmas with 2,7-dichlorodihydrofluorescein diacetate (H_2_DCFDA). The results revealed a gradual accumulation of ROS in the stigma from S1 to S2 stage, followed by an increase of approximately 7-fold in the S3 stage (Fig. 5a). Additionally, aniline blue staining was used to explore the role of ROS in pollination. Pollen attachment was observed in the mature stigma at pollination, with fewer pollen grains sticking to the stigma and failing to germinate at 30 min after pollination (30 MAP). Pollen attachment increased significantly at 60 min after pollination (60 MAP), with partial germination of pollen. At 120 min after pollination (120 MAP), all the pollen tubes germinated and grew toward the style (Fig. 5b).

H_2_DCFDA staining was employed to detect ROS levels in the stigma after pollination (0, 30, 60, and 120 MAP). Compared to the ROS level at 0 MAP, a significant reduction in the ROS level was observed at 30 and 120 MAP (Fig. 5c). These results indicate that ROS levels increase during stigma development, but decrease after pollination and pollen tube growth, suggesting ROS impact stigma development, pollination, and pollen tube growth.

### Reduced ROS levels accelerate pollen number and pollen tube growth in the mature stigma

**Figure 6:**
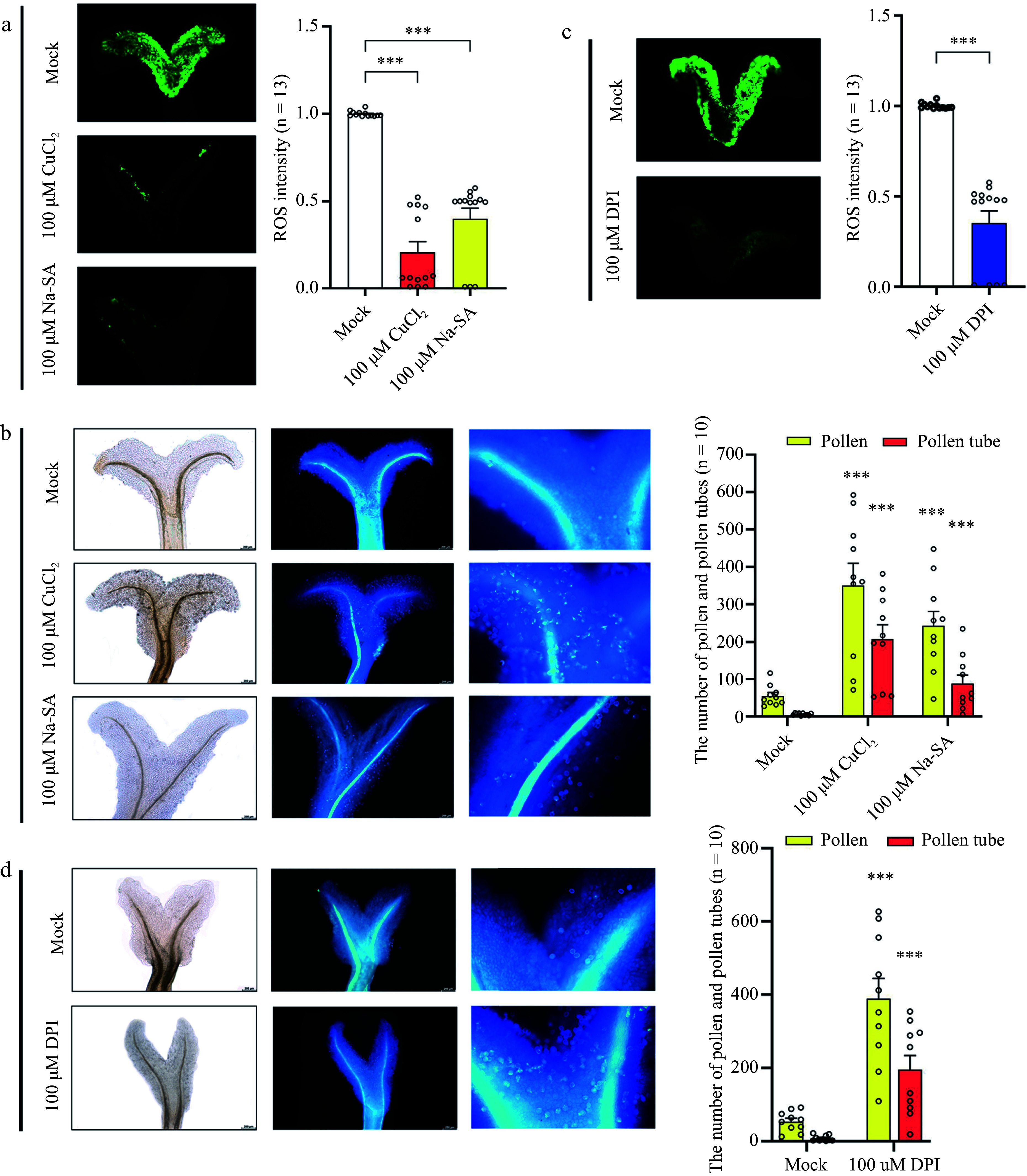
Effect of ROS sequestration on pollination. (a) Detection of ROS after treatment of mature stigmas with copper (II) chloride (CuCl_2_) and sodium salicylate (Na-SA). (b) Detection of pollen number and pollen tube number after stigmas were treated with CuCl_2_ and Na-SA. From left to right: bright field and ultraviolet light views. (c) Detection of ROS after treatment of mature stigmas with diphenyleneiodonium chloride (DPI). (d) Detection of pollen tube number after mature stigmas were treated with DPI. From left to right: bright field and ultraviolet light views. Data are expressed ± SEM. Each data point is indicated by a dot, and (n) denotes the number of stigmas. Asterisks or not significant (ns) directly above the data bars indicate significance level compared with the data bar on the far left (Student's t-test; ***p < 0.001).

To investigate whether reduced ROS levels in the mature stigma could increase pollen number and pollen tube germination, mature stigmas were treated with ROS scavengers, copper (II) chloride (CuCl_2_) and sodium salicylate (Na-SA) (Fig. 6a). Additionally, an inhibitor of NADPH oxidases, diphenyleneiodonium chloride (DPI), was used to suppress ROS levels in mature stigmas (Fig. 6c). The findings revealed that 30 min after pollination, there was a significant increase in both the number of pollen and pollen tubes on the stigma compared to the mock-treated group (Fig. 6b, d). The results indicate that ROS scavengers and NADPH oxidase inhibitors can effectively reduce ROS levels in the mature stigma. Furthermore, the results demonstrate an enhancement in pollen tube germination on the stigma, suggesting a potential association between the reduction in ROS levels and the induction of pollen–stigma interactions.

## Discussion

Stigma development and pollination are important factors for successful reproduction in flowering plants. Studies in various plant species have revealed the usefulness of transcriptome data in deciphering the mechanisms underlying stigma development and pollination^[26,27]^. Despite the widespread distribution of *F. mandshurica* across northeast China, Russia, and Europe, few studies investigate the biology of its floral organs^[28]^. In our study, the RNA-seq data revealed 6,402 DEGs and 3,460 transcription factors (TFs) across the developmental stages (S1 vs S2, S2 vs S3, and S1 vs S3 libraries), which included ROS synthesis genes, antioxidant enzymes, and flavonoid synthesis genes. In general, *F. mandshurica* female plants are more sensitive to stress^[28]^, leading to increased ROS production and enhanced metabolic activity. Functional annotation of these DEGs highlighted their involvement in diverse biological pathways, including plant–pathogen interactions, as well as flavonoid and carotenoid synthesis. Moreover, the evolutionary perspective proposes that pollination has emerged from interactions between plants and pathogens^[29,30]^, which leads to the accumulation of ROS and the activation of scavenging systems, including antioxidant enzymes and non-enzymatic small molecules such as glutathione, flavonoids, and carotenoids^[31]^. Therefore, global transcriptional profiles enable researchers to study the mechanisms underlying stigma development, and this information can greatly advance our understanding of plant reproductive biology.

Reactive oxygen species exist in many forms within cellular environments, with the major forms including H_2_O_2_ (hydrogen peroxide),^1^O_2_ (singlet oxygen), O_2_^·−^ (superoxide), HO^−^ (hydroxyl radical), and other organic and inorganic peroxides^[31−33]^. Our analysis of the RNA-seq data revealed changes in the expression of genes related to ROS production during stigma development. Specifically, three differentially expressed unigenes encoding RBOH proteins (*RBOHA-1, RBOHA-2*, and *RBOHC*) were identified. RBOH proteins regulate the activity of ROS-producing NADPH oxidase (NOX), whose activation is triggered by Ca^2+^-dependent phosphorylation and Ca^2+^ binding to EF-hand motifs^[34]^. For example, *St*CDPK proteins have been reported to stimulate NADPH oxidase via phosphoric acid, which increases the production of ROS^[35]^. In our study, both *CNGCs* and *CDPKs* were highly expressed during S2 and S3 stages, aligning with the expression trends of *ROBH* genes, suggesting Ca^2+^ can modulate *RBOH* activity during stigma development. Furthermore, other enzymes can contribute to ROS production. For example, AO not only regulates aldehyde oxidation but also influences NADPH oxidase activity, leading to the generation of superoxide anions^[36,37]^. Additionally, NADPH oxidase and PAO synergistically regulate the accumulation of H_2_O_2_ and superoxide (O_2_^·−^) in tobacco^[38,39]^. To sum up, these genes regulate ROS production during stigma development. The activation of *CDPK* and other genes may lead to the activation of downstream gene *RBOH*. In the β-oxidation pathway, ACOX converts acyl-CoA into trans-2-enyl-CoA while producing H_2_O_2_ as a by-product^[40]^. Similarly, SOX covalently binds FAD molecules, facilitating the decomposition and metabolism of lysine, thereby producing H_2_O_2_ as a by-product^[41]^. Apart from the elevated expression of *RBOH* genes during the S2 and S3 stages, we also observed the activation of several transcripts encoding AO, SOX, PAO, and ACOX in the S2 stage and their increased expression across the three stages of stigma development. Taken together, these findings suggest that these genes act synergistically to promote the accumulation of ROS during stigma development.

Low levels of ROS are critical in plants, protecting cells from damage. When ROS levels increase, antioxidant mechanisms are activated to sequester ROS^[32]^. Our study identified several enzymes, including SOD and PRX, which play important roles in defending plants from the damaging effects of ROS. These enzymes catalyze the dismutation of O_2_^·−^ to H_2_O_2_ and O_2_ in mitochondria, chloroplasts, peroxisomes, and apoplasts^[31,41−43]^. Monodehydroascorbate reductase (MDAR), another antioxidant enzyme, can also scavenge ROS^[44]^. The current study revealed consistent expression trends among genes encoding SOD, PRX, MDAR, TRX, GPX, GST, and GRX, indicating ROS production activates antioxidant enzyme expression. During the S2 stage, the levels of RBOH, AO, and PAO were upregulated in both the apoplast and cytoplasm, suggesting that ROS content in the stigma changed during the S2 stage, which activated the ROS scavenging system in the stigma.

Flavonoid synthesis directly impacts various physiological processes that are crucial for floral organ growth and development^[45,46]^. Elevated levels of flavonoids have been reported in both the stigma and pollen^[47]^, with the key components of the flavonoid synthesis pathway (PAL, CHS, F3H, and FLS) regulating flavonoid accumulation in plants^[48]^. In ornamental kale, flavonoids act as antioxidants and alleviate self-incompatibility, with four flavonoid-producing genes (*CHS1*, *CHS2*, *F3H*, and *FLS*) consistently expressed in the stigma^[49]^. Flavonoids also facilitate reproduction in plants under harsh environmental conditions^[50]^ and regulate stigma development by interacting with components of the ROS synthesis pathway^[51−53]^. They are also involved in pollination, with studies suggesting opposing roles for flavonoids and ROS^[49]^. Flavonoids and ROS are also abundant during gametophyte development^[47,50]^.

Chalcone synthase (CHS) is abundant in both the stigma and pollen of *Petunia*, and mutations in the *CHS* gene lead to defective stigma and sterility^[54]^. Flavonoids also regulate pollen and stigma interactions in *Arabidopsis* and *Helianthus annuus*^[47,55]^. Consistent with prior research, increased expression of PAL-1, CHS, F3H-1, and FLS in the S2 and S3 stages of stigma development were observed, and their expression patterns aligned with those of ROS-related genes, suggesting flavonoids participate in stigma development and pollination in *F. mandshurica*.

High levels of ROS impact various aspects of plant biology, including stress response, stigma development, pollen development and release, and pollen tube growth^[15,31,56]^. Interestingly, ROS accumulation in flowers has been reported to be critical for plant reproduction^[49,57]^. During this process, ROS control pollination, with ROS functioning as key determinants of the plant's decision-making process for successful fertilization^[58]^. After pollination, ROS levels decrease in the stigma, while incompatible pollination brings about increases in ROS levels, thus leading to self-incompatibility^[59,60]^. For example, FERONIA (FER), an important determinant of self-incompatibility, is regulated by ROS to ensure pollen growth and germination^[60]^.

In the present study, ROS levels in the stigma were assessed by H_2_DCFDA staining. It was observed that ROS accumulated in all stages of stigma development, reaching peak levels during the S3 stage. The accumulation of ROS in the stigma not only promotes successful pollination but also mediates pollen germination and growth through a decrease in ROS levels^[61]^, consistent with prior research reporting a reduction in ROS levels after pollination^[49,60]^. In tobacco plants, the reduction of H_2_O_2_ in the stigma accelerates pollen germination and guarantees successful reproduction^[62]^. Other ROS, including ·O^2−^, ·OH, and OH^–^, also participate in the interactions between pollen and stigma during reproduction^[63]^.

This is the first study to investigate the role of ROS scavengers in pollen–stigma interactions in *F. mandshurica*. The presence of ROS production and sequestration mechanisms in the reproductive tissues of plants has been reported in various species^[64]^, and the accumulation of ROS in the stigmas of angiosperms is well documented^[65]^. The interactions between pollen and stigma are important for pollen attachment, hydration, adhesion, and germination. Furthermore, the content of ROS in stigma increased significantly after incompatible pollination and decreased after compatible pollination, elevated ROS levels in stigma can inhibit the germination of compatible pollen, suppressing RBOH or FERONIA(FER) receptor kinase homolog or Rac/Rop guanosine triphosphatase (GTPase) which can effectively reduce the content of ROS in stigma and cause incompatible pollen to germinate^[60]^. Proteins and lipids found on the surface of pollen are involved in this process. In *Arabidopsis*, KINβγ mediates ROS production in mitochondria, which is essential for pollen hydration and germination^[66]^. Common ROS scavengers, such as CuCl_2_, Na-SA, and DPI, along with NADPH oxidase inhibitors, have been used to sequester ROS, resulting in enhanced pollen attachment^[59,60]^. Other studies have demonstrated that ROS inhibit pollen adhesion and germination^[61]^, and a reduction in ROS levels in the stigma before pollen attachment is critical for pollen–stigma interactions and pollen germination^[67]^. As for DPI and catalase, both scavengers enhance pollen germination in cucumber by inhibiting NADPH oxidase activity^[68]^. Similarly, DPI decreases ROS levels, thus promoting pollen adhesion and germination^[69]^. Taken together, these findings underscore the role of ROS accumulation in stigma development, highlighting the importance of reducing ROS for pollen attachment and germination.

## Conclusions

This study utilized RNA-seq data to identify crucial genes involved in both the production and sequestration of ROS during stigma development in *F. mandshurica*. Our results underscore the pivotal role played by ROS in regulating stigma development, pollination, and pollen tube growth. Staining revealed ROS accumulation during stigma development, and when stigmas were treated with ROS scavengers and an NADPH oxidase inhibitor, there was a decrease in ROS levels and an increase in pollen adhesion and germination. Taken together, these findings greatly contribute to our comprehension of reproduction in *F. mandshurica*.

## SUPPLEMENTARY DATA

Supplementary data to this article can be found online.

## Data Availability

The datasets generated during and/or analyzed during the current study are available from the corresponding author on reasonable request.
