# Supplementary material for: Transcriptomics reveal the involvement of reactive oxygen species production and sequestration during stigma development and pollination in Fraxinus mandshurica
Source: For Res (Fayettev). 2024 Apr 23;4:e014. doi: 10.48130/forres-0024-0011 (PMC11524289; doi:10.48130/forres-0024-0011)
Supplement: Supplementary file 1 — Supplementary data to this article can be found online. [file forres-0024-0011-S1.zip › 10.48130_forres-0024-0011-Suppl-TableS1.pdf]

Supplemental table S1: Raw and clean reads of 9 RNA samples of *Fraxinus mandshurica* stigma

Sample	Total Raw Reads (M)	Total Clean Reads (M)	Clean Base(G)	Q20(%)	Q30(%)	GC Content(%)
**S1-1**	80.49	77.05	11.56	98.84	95.22	43.51
**S1-2**	90.44	86.85	13.03	99.03	95.95	43.56
**S1-3**	82.23	78.87	11.83	98.91	95.48	43.47
**S2-1**	80.77	77.71	11.66	98.86	95.29	43.22
**S2-2**	88.62	82.25	12.34	98.88	95.38	43.36
**S2-3**	74.23	70.94	10.64	98.83	95.21	43.23
**S3-1**	80.57	77.02	11.55	98.91	95.5	43.4
**S3-2**	80.92	76.84	11.53	98.94	95.66	43.38
**S3-3**	78.26	74.24	11.14	98.81	95.1	43.44
