# Supplementary material for: Transcriptomics reveal the involvement of reactive oxygen species production and sequestration during stigma development and pollination in Fraxinus mandshurica
Source: For Res (Fayettev). 2024 Apr 23;4:e014. doi: 10.48130/forres-0024-0011 (PMC11524289; doi:10.48130/forres-0024-0011)
Supplement: Supplementary file 1 — Supplementary data to this article can be found online. [file forres-0024-0011-S1.zip › 10.48130_forres-0024-0011-Suppl-FigureS1.pdf]

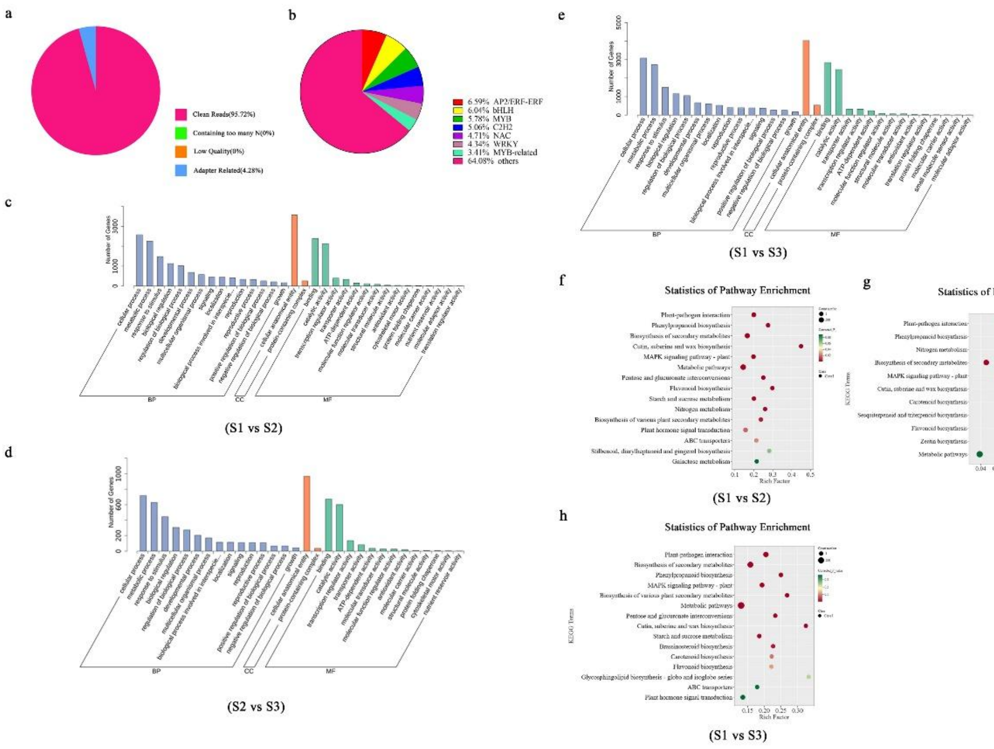


Supplemental Fig. S1: Transcriptome analysis of different developmental stages of stigma of Fraxinus mandshurica. (a) Transcriptome data quality statistics. (b) Transcription factor statistics. (c) GO enrichment analysis of S1 vs S2 (d) GO enrichment analysis of S2 vs S3. (e) GO enrichment analysis of S1 vs S3. (f) KEGG enrichment analysis of S1 vs S2. (g) KEGG enrichment analysis of S2 vs S3. (h) KEGG enrichment analysis of S1 vs S3.
